# A mechanical model for predicting the probability of osteoporotic hip fractures based in DXA measurements and finite element simulation

**DOI:** 10.1186/1475-925X-11-84

**Published:** 2012-11-14

**Authors:** Enrique López, Elena Ibarz, Antonio Herrera, Jesús Mateo, Antonio Lobo-Escolar, Sergio Puértolas, Luis Gracia

**Affiliations:** 1 Department of Design and Manufacturing Engineering, University of Zaragoza, Zaragoza, Spain; 2 Department of Mechanical Engineering, University of Zaragoza, Zaragoza, Spain; 3 Department of Surgery, University of Zaragoza, Zaragoza, Spain; 4 Department of Orthopaedic Surgery and Traumatology, Miguel Servet University Hospital, Zaragoza, Spain; 5 Aragón Health Sciences Institute, Zaragoza, Spain; 6 Engineering and Architecture School, University of Zaragoza, María de Luna, 3, 50018, Zaragoza, Spain

**Keywords:** Osteoporosis, Osteoporotic fracture, Predictive model, Finite elements, Fracture risk, Fracture probability

## Abstract

**Background:**

Osteoporotic hip fractures represent major cause of disability, loss of quality of life and even mortality among the elderly population. Decisions on drug therapy are based on the assessment of risk factors for fracture, from BMD measurements. The combination of biomechanical models with clinical studies could better estimate bone strength and supporting the specialists in their decision.

**Methods:**

A model to assess the probability of fracture, based on the Damage and Fracture Mechanics has been developed, evaluating the mechanical magnitudes involved in the fracture process from clinical BMD measurements. The model is intended for simulating the degenerative process in the skeleton, with the consequent lost of bone mass and hence the decrease of its mechanical resistance which enables the fracture due to different traumatisms. Clinical studies were chosen, both in non-treatment conditions and receiving drug therapy, and fitted to specific patients according their actual BMD measures. The predictive model is applied in a FE simulation of the proximal femur. The fracture zone would be determined according loading scenario (sideway fall, impact, accidental loads, etc.), using the mechanical properties of bone obtained from the evolutionary model corresponding to the considered time.

**Results:**

BMD evolution in untreated patients and in those under different treatments was analyzed. Evolutionary curves of fracture probability were obtained from the evolution of mechanical damage. The evolutionary curve of the untreated group of patients presented a marked increase of the fracture probability, while the curves of patients under drug treatment showed variable decreased risks, depending on the therapy type.

**Conclusion:**

The FE model allowed to obtain detailed maps of damage and fracture probability, identifying high-risk local zones at femoral neck and intertrochanteric and subtrochanteric areas, which are the typical locations of osteoporotic hip fractures.

The developed model is suitable for being used in individualized cases. The model might better identify at-risk individuals in early stages of osteoporosis and might be helpful for treatment decisions.

## Background

Currently, osteoporotic fractures represent major cause of disability, loss of quality of life and even death among the elderly population [1]. Osteoporosis is caused by a skeletal involution linked to aging, which is more prevalent in women: the lifetime risk for a fragility fracture at the age of 50 lies within the range of 40% in women [2].

Hip fracture is considered the most devastating osteoporotic fracture. It generally occurs around age 80 [3] and affects women more often than men in a ratio 3:1 [4]. The worldwide incidence of hip fractures in 2000 was estimated at 1.6 million [5] and, if present population forecasts are borne out, there will be 6.26 million in 2050 [6]. Hip fracture is now a major cause of morbidity, mortality and disability, and represents a significant economic cost [4,5,7,8].

Decisions on drug therapy in osteoporotic patients without previous fractures are mainly based on the analysis of risk factors which predispose to fracture. A risk assessment tool called FRAX® (Fracture Risk Assessment Tool) has been developed by the World Health Organization (WHO) for this purpose [9-12]. The risk of major osteoporotic fractures (hip, vertebrae, humerus and wrist), or specifically hip fracture over the next 10 years can be estimated with the FRAX® tool. The probability of fracture is calculated on the basis of age, body mass index and several dichotomized variables (previous fracture, smoking, rheumatoid arthritis, etc.). Optionally, bone mineral density (BMD) of the femoral neck can be included for risk calculation. Other studies have questioned the effectiveness of FRAX as a tool for predicting fracture risk [13,14].

Several previous surveys have assessed the risk of fracture using various methodologies, but mostly based on BMD measurements [15,16]. BMD measurements have also been used for determining the mechanical strength [17] or to develop statistical models for predicting the risk of fracture [18]. Another study used morphological measurements of the femoral head and neck to determine the risk of fracture [19].

Regarding simulation by means of finite element method, both micro and macro-mechanical models have been suggested, with different characteristics and methodologies. These models can be used for prediction of bone strength at different ages, or to prediction of fracture risk. Thus, Lee [20] provides a micromechanical model of bone behavior under different densities, which is difficult to extrapolate to the scale required to get realistic predictions. Boccacio, Zhang [21,22] provide more advanced macro-mechanical models, which analyze a complete functional unit of the spine (two vertebrae with their intervertebral disk) in terms of mechanical behavior depending on bone density. Macneil [23] sets a 2D model in the sagittal plane (vertebrae L1-L4), using bone geometry and BMD measurements obtained from radiographs and DXA. In this model, developed for the analysis of vertebral fractures, the stiffness is calculated based on the patient's age, taking into account an exponential decline. In the case of proximal femoral fractures, Kaneko [24] develops a model based on imaging techniques (Quantitative Computed Tomography (QCT)), focused on the estimation of bone strength as a function of age in normal populations. The model aimed to establish a statistical correlation between the prediction of bone strength and the risk of osteoporotic fracture. A different methodology is used by Bryan [25], who suggests a parametric model incorporating both the geometry and the properties of bone, which allows a range of results reflecting the statistical variation of the model parameters. A similar methodology is used by Bessho [26], although the parametric analysis, in this case, refers to load and support conditions of the model. Some authors have begun to incorporate yield criteria for fracture risk prediction. In this respect, Derikx [27] applies the Drucker-Prager criterion on a model made from QCT, with asymmetric yielding in tension and compression. Similarly Tellache [28] applies an anisotropic yield criterion on a model constructed from imaging (CT scan) for prediction of fracture risk. With a different approach, Amin [29] performed a comparative analysis of fracture risk predictions based on BMD measurements against those ones obtained from an FE model developed from QCT, correlating bone strength with fracture risk. Finally, on the matter of drug treatments, Keaveny [30] discusses the influence on bone strength of PTH and alendronate, using a FE model developed from QCT scans of osteoporotic patients. Currently, the most popular tool to assess the fracture risk is FRAX, combining different clinical factors and BMD measurements. However, all the above methods have some limitations concerning their approaches, including clinical or mechanical magnitudes related to bone fracture in an independent way, but without consider their mutual influence as actually happens. So, clinical BMD values allow evaluating the mechanical properties of bone (stiffness and resistance), which condition the stresses patterns causing cumulative mechanical damage. That cumulative damage would cause fractures in different load scenarios.

In view of the current models limitations, we have developed a model for predicting the risk of osteoporotic hip fractures based on the Damage Mechanics and Fracture Mechanics. This model will be incorporated into a finite element code to simulate their evolution over time. Thus, we will estimate the probability of hip fracture in the mid and long terms. Bone mineral density (BMD) measurements, from dual energy X-ray absorptiometry (DXA) scans, will be incorporated into the model in order to fit it to clinical conditions. The model is not intended for simulating the bone fracture, but to predict the degenerative process in the skeleton, with the consequent lost of bone mass and hence the decrease of its mechanical resistance which enables the fracture due to different traumatisms.

## Methods

In order to develop a predictive model which takes into account the mechanical parameters involved in the fracture process, a correlation between these magnitudes and those ones measured in clinical terms is firstly required. To this effect, Carter and Hayes [31] established a direct relationship between Young's modulus and bone density for low strain rates (0.01):

(1) $$E=2875\;\rho^{3}$$

On the other hand, the relationship between the BMD value and the apparent density is adjusted, according to experimental results, as:

(2) $$\rho =\rho_{\mathrm{máx}}{\left(\frac{BMD}{BMD_{\text{máx}}}\right)}^{\lambda }$$

being λ a parameter that depends on the sample data. For the present study a value of 9/25 was assigned for λ, in order to fit the actual data presented in [32].

BMD is the current standard for diagnosis of osteoporosis. Over 100 worldwide published papers assessing hip BMD evolution, both in natural conditions and in patients under drug therapy were selected for analysis. Among them, three treatments have been selected for the comparative study: alendronate 10 mg per day [33], oral ibandronate 2.5 mg per day [34] and PTH 1–84 100 mg per day [35]. These drugs have proven to be effective, and their BMD evolution curves show the required regularity in the analyzed time period.

Bisphosphonates settle in the bone tissue and its effect persists during some time after its administration, and hence it is completely accepted in the clinical practice the use of intermittent or discontinuous treatments. In most of the studies a maximum five years follow-up is done, although a treatment continuous for ten years is also accepted. Moreover, there are several studies concerning the safety of long term treatments (ten years) [36-42]. With respect to the use of combined therapies (teriparatide and bisphosphonates), it is a usual clinical practice in the osteoporosis treatment [43-47].

Regarding the natural history of BMD, the average curve published by Mazess [48] was chosen as a reference. Briefly, the natural curve of BMD linked with age was compared to the curves of BMD in patients treated with bisphosphonates or derivatives of parathyroid hormone.

Since the standard adjustment techniques do not provide enough accuracy and reliability to be applied to the predictive model, higher level, more complex, adjustments have been set out to obtain continuous curves of regression. These make it possible to obtain continuous curves for the BMD evolution. The evolution trends of the different selected cases in the study can be extrapolated from those continuous curves, ensuring a consistent behavior. The following adjustments were proposed:

Polynomial:

(3) $$\rho =a_{n}t^{n}+a_{n-1}t^{n-1}+\ldots +a_{1}t+a_{0}$$

Exponential:

(4) $$\rho =k\left(1-e^{-\left(a_{n}t^{n}+a_{n-1}t^{n-1}+\ldots +a_{1}t+a_{0}\right)}\right)$$

Exponential asymptotic:

(5) $$\rho =\rho_{0}+\left(\rho_{\mathrm{lím}}-\rho_{0}\right)\left(1-e^{-\frac{at}{t_{m}-t}}\right)$$

**Figure 1:**
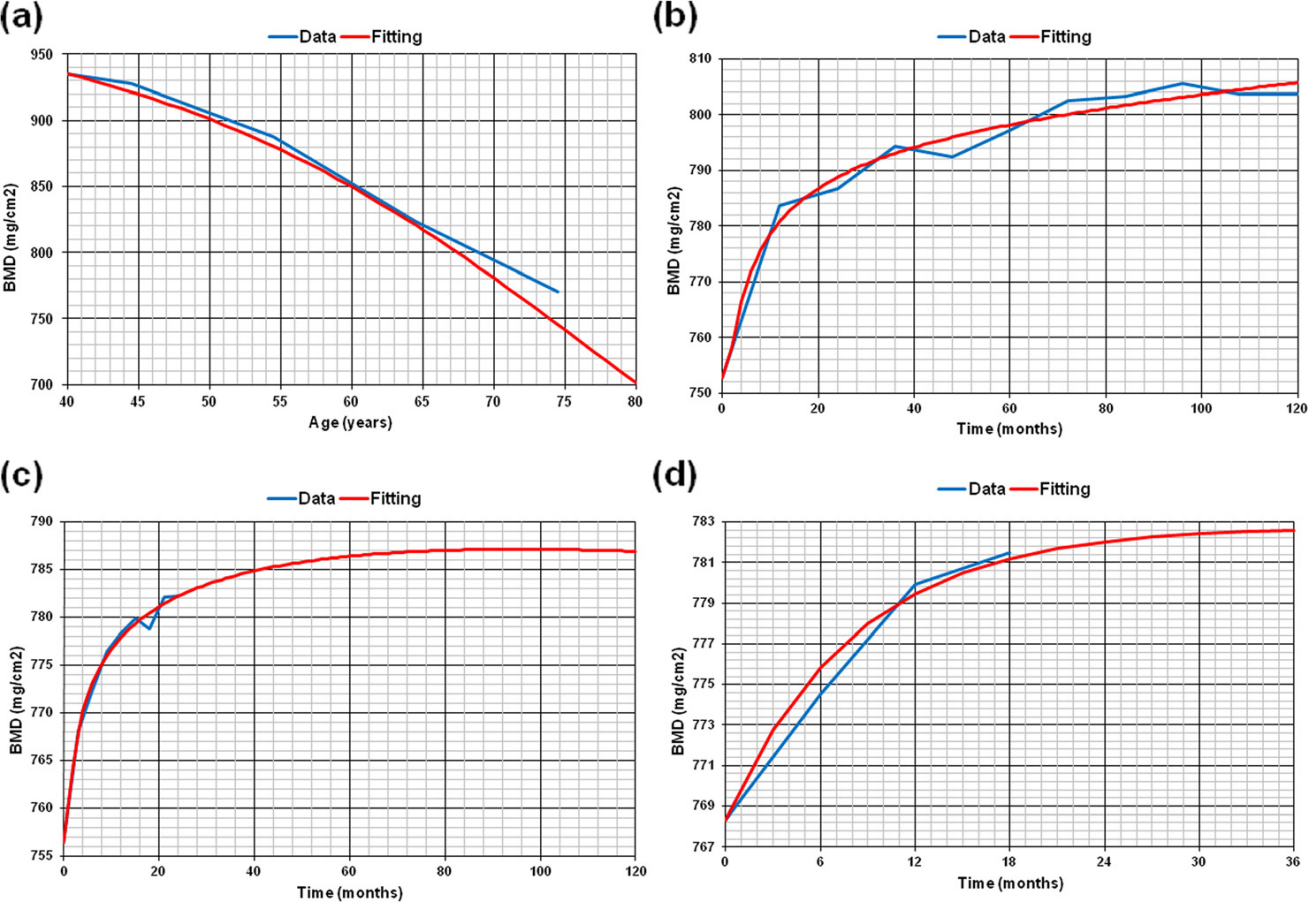
**Fitting of BMD evolutionary curves: a) Natural evolution (R**^**2**^** = 0.992); b) Alendronate (10 mg/day) (R**^**2**^** = 0.980); c) Oral ibandronate (2,5 mg/day) (R**^**2**^** = 0.991); d) PTH 1–84 (100 mg/day) (R**^**2**^** = 0.982).**

These adjustments (Equations 3, 4 and 5) have been applied to the four examined curves (natural evolution and the three therapies). Several choices have been made during this adjustment: the lowest mean square error, the closest to unity correlation coefficient R^2^, and a 10-year standardized follow-up period with the possibility of extrapolation to longer periods up to 15 years (Figure 1). That extrapolation is intended to be consistent in mathematical terms, independently of the actual duration of the considered study. This condition will permit in the future an easy incorporation of more long term studies when available. Standardization of BMD measurements included in each published paper was required in order to make comparisons among them [49], because de data were obtained from different densitometric equipment depending on the study (Hologic, Lunar and Norland).

The Karganovin^′^s Damage Mechanics model [50] was selected for the simulation of the degenerative process. This model defines the mechanical damage, *D*, as a function of the equivalent strain, *ε*_*c*_:

(6) $$D=1-\kappa {\left(\varepsilon_{c}\right)}^{\gamma }$$

being:

(7) $$\varepsilon_{c}=\sqrt{\varepsilon_{I}^{2}+\varepsilon_{\mathrm{II}}^{2}+\varepsilon_{\mathrm{III}}^{2}-\varepsilon_{I}\varepsilon_{\mathrm{II}}-\varepsilon_{I}\varepsilon_{\mathrm{III}}-\varepsilon_{\mathrm{II}}\varepsilon_{\mathrm{III}}}$$

the equivalent strain, where *ε*_*I*_, *ε*_*II*_, *ε*_*III*_ are the principal components of the strain tensor. This mechanical damage leads to a decrease of the bone stiffness, according with the relationship:

(8) $$D=1-\sqrt{\frac{E}{E_{0}}}$$

where *E*_*0*_ corresponds to the Young’s modulus of healthy bone and *E* is the actual value of Young’s modulus of the bone with cumulative mechanical damage. The *κ* and *γ* constants depend on the critical damage value, the critical strain and the strain threshold, *ε*_*0*_, below which no damage is produced (*ε*_*c*_ = *ε*_*0*_ implies *D* = *0*):

(9) $$\begin{array}{cccc} \gamma =\frac{\ln\left(\frac{1}{1-D_{\mathrm{cri}}}\right)}{\ln\left(\frac{\varepsilon_{0}}{\varepsilon_{\mathrm{cri}}}\right)} & & & \kappa =\frac{1}{{\left(\varepsilon_{0}\right)}^{\gamma }} \end{array}$$

where *ε*_*cri*_ corresponds to the equivalent strain value that produces the critical damage, *D*_*cri*_. Once the damage model is defined, a relationship between the damage level and the probability of fracture must be set. Obviously several factors are involved in the equation and may difficult the calculations: damage location, amount of damage, range and type of load cycles, and so on. However, since osteoporosis is a generalized disease affecting bone mass extensively, and it occurs in older people with little variation in their life habits, a simplified model could be used. In this work, a model of fracture probability based on the law of Paris [51], which explains the stable crack growth under monotonic loading, was developed. The number of loading cycles needed to increase damage from the *D*_*i*_ value to the *D*_*f*_ value was expressed as follows:

(10) $$N=\frac{1}{\alpha \;{\left(\Delta \sigma \right)}^{\beta }\gamma^{\beta /2}\left(\frac{\beta }{2}-1\right)}\left[{\left(\frac{1}{\omega D_{i}}\right)}^{\frac{\beta }{2}-1}-{\left(\frac{1}{\omega D_{f}}\right)}^{\frac{\beta }{2}-1}\right]$$

where *α* and *β* are material parameters associated with the law of Paris, *γ* is a parameter related to the stress intensity factor defined in Linear Elastic Fracture Mechanics, which depends on geometric aspects and stress distribution, Δ*σ* is the variation of stress in each loading cycle and *ω* is a parameter which relates the mechanical damage to the crack size *a* (*a* = *ωD*).

By normalizing the probability of fracture, assigning value 1 to critical damage (*D* = *D*_*cri*_)_,_ and value 0 to no damage (*D* = *0*), we finally obtain the probability of fracture as a function of both the number of cycles and the damage:

(11) $$P\left(fracture\right)=1-\frac{N\left(D\right)}{N_{\max}}={\left(\frac{D}{D_{\mathrm{crí}}}\right)}^{\frac{\beta }{2}-1}$$

In Equation (11), the magnitude *N*_*max*_ represents the maximum number of load cycles necessary for a mechanical damage equivalent to the critical value, *D*_*cri*_. A value *5* has been given to the *β* coefficient for cortical bone, according to Taylor [52], and, in accordance with Kargarnovin [50], a critical damage, *D*_*cri*_, of *0*.*38* and a critical strain, *ε*_*cri*_, of *0*.*0174* have been considered, with a strain threshold, *ε*_*0*_, of *0*.*0015* (no damage is produced by strains below *0*.*0015*).

Progression curves are basic references and provide only general information. In order to apply the model to specific patients we must consider, in addition to the trend, the reference density value of the patient. According to the law of interpolated natural evolution, the density matching with the age of the patient is given by:

(12) $$\rho_{N}\left(t_{0}\right)\neq \rho_{0}$$

being *ρ*_*N*_(*t*_*0*_) the average density measured in the patient and *ρ*_*0*_ the matching value from the reference progression curve, that is, the actual patient’s density doesn’t necessarily be equal to the value corresponding to the average curve for the considered population. There is an offset that should be added to the reference progression curve of density, providing a translation of the average curve, allowing an adaptation for each individual patient, so:

(13) $$\rho_{p}^{N}\left(t\right)=\rho_{N}\left(t\right)+\left[\rho_{0}-\rho_{N}\left(t_{0}\right)\right]$$

When a drug therapy is applied to the same patient, a similar correction is required because a new offset arises. So, the progression curve for this patient under a treatment would be:

(14) $$\rho_{p}^{T}\left(t\right)=\rho_{T}\left(t\right)+\left[\rho_{0}-\rho_{T}\left(t_{0}\right)\right]$$

In the Equations (12) to (14), the subscripts or superscripts *N* and *T* represent natural evolution or evolution with treatment, respectively. All these adjustments provide the estimated BMD value for any type of patient, at any age, and under any prescribed therapy. From this value, mechanical properties of bone can be calculated. It must be noticed that the considered curves represent the mean evolutionary curves for the population, and an individual patient could not follow the curve exactly but in an approximate way.

Densitometric data of the healthy femur have been taken as the starting point for this study, based on a previous work of our group on the biomechanical behavior of a femoral stem [32]. In that survey, densitometric data were correlated both with the apparent volumetric density, and with the Young moduli of each of the Gruen zones. Table 1 depicts the BMD, the apparent volumetric density, and the Young moduli correlation corresponding to a healthy femur.

**Table 1:** **BMD**, **apparent density and Young**’**s modulus for the data corresponding to the study of Herrera**[40]

**GRUEN Zone**	**BMD Hologic (mg/cm**^**2**^**)**	**BMD standarized (mg/cm**^**2**^**)**	**Apparent density(gr**/**cm**^**3**^**)**	**Young**’**s modulus(MPa)**
1	782	794	1,480	9287
2	1093	1108	1,669	13333
3	1429	1446	1,837	17810
4	1591	1610	1,909	20000
5	1530	1548	1,882	19173
6	1302	1318	1,777	16107
7	1192	1208	1,721	14642

**Figure 2:**
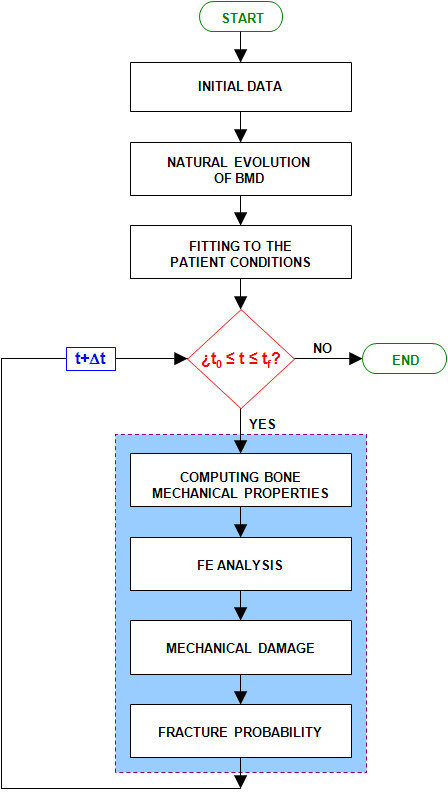
Evolutionary algorithm for the prediction of fracture probability.

**Figure 3:**
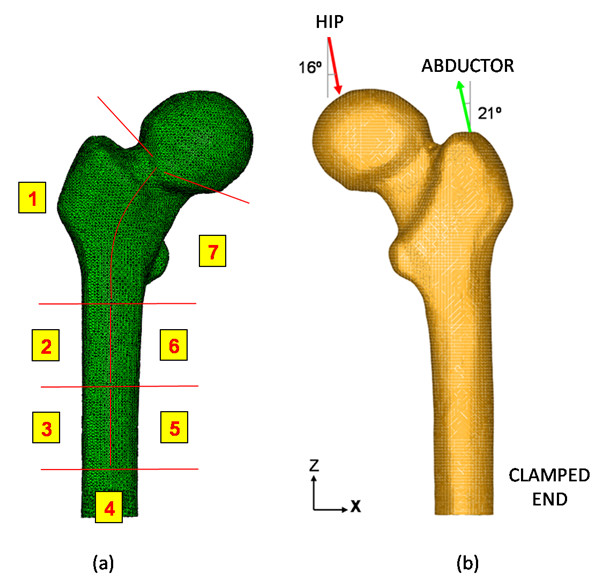
3D FE model of proximal femur: a) Adaptation of Gruen zones to the healthy femur; b) Boundary conditions on the proximal femur.

From all the previous calculations, an evolutionary algorithm has been implemented (Figure 2), which has been used combined with a finite element model of the femur [32] using the Abaqus software [53]. The procedure was based on a finite element model of the upper half of the femur, made up of tetrahedral elements with quadratic approximation (C3D10 in Abaqus nomenclature), as shown in Figure 3A and b. To generate the model a 3D laser scanner Roland PIZCA was used for the outer geometry and thirty transverse direction tomographic cross-sections and eight longitudinal direction cross-sections were taken using CAT (General Electric Brightspeed Elite) to determine the geometry of the cancellous bone. Moreover a 3D reconstruction was made to obtain the shape of the medullar cavity [32].

In the final mesh, consisting of 408094 elements and 75223 nodes, cortical bone, cancellous bone and bone marrow have been differentiated (229931 elements for cortical bone, 166220 elements for cancellous bone and 11943 elements for bone marrow). To guarantee the accuracy in the FE results, a sensitivity analysis was performed with a mesh refinement in order to achieve a convergence towards a minimum of the potential energy, with a tolerance of 1% between consecutive meshes.

In addition, the femur has been divided into 7 zones of Gruen [54], since the available information on BMD was related to these zones (Figure 3A). The mechanical properties obtained from the evolutionary algorithm have been assigned to each of the Gruen zones. For this purpose, the apparent density is calculated for each Gruen zone from its BMD measure according Equation (2), and then, the Young’ modulus is obtained from Equation (1). The same process was followed for cancellous bone. Any case, cortical bone stresses are slightly influenced by the mechanical properties of cancellous bone and bone marrow since their stiffness is much lower than the corresponding to cortical bone. The function of cancellous bone, in mechanical terms, consists in providing stability to the thin walled cortical bone. A linear elastic behavior has been defined for all the materials. The final collection of results was focused on the proximal femur, mainly on the area located within the edge of the femoral head and the subtrochanteric region.

As a boundary condition, the middle third of the femoral diaphysis was clamped (Figure 3B), since this area is far enough from the proximal femur to avoid significant perturbations in the stress distribution. Thus, computational cost was reduced if compared with the entire femur model.

In the finite element model construction, as important as the reaction strength on the femoral head due to the body weight, is the inclusion of the muscle forces to be considered in the simulation. In our model only the abductor muscle forces were included, in line with several authors [55,56]. As a rule of thumb, abductor muscles cause a reaction on the femoral head of 2.75 times the body weight. However, load increases as much as 4 times the body weight when the heel impacts the ground, and during the double support stage of the gait [57]. The latter situation was considered, as the worst case, in order to set the boundary conditions. According to data from the study [32] a 79.3 kg body-weight was set as a reference. So, two load conditions were imposed (Figure 3B):

• Reaction strength on the femoral head due to the body weight (3110 N).

• Load due to the abductor muscles, applied to the proximal area of the greater trochanter (1360 N).

The above values are slightly higher than those included in recent studies [58,59]. This model has been used in predicting the evolution of femoral fracture probability, by comparing the natural history and the expected evolution under different therapies.

## Results

Firstly, adjustment models were applied both to BMD physiological curve (weighted average from [48]) and the curves of patients under three different therapies: alendronate (10 mg per day), oral ibandronate (2.5 mg per day) and PTH 1–84 (100 mg per day). Figure 1 shows the different evolutionary curves obtained in each case, together with the interpolated curves. Despite an extreme irregularity of some values, the correlation coefficients have been 0.992, 0.980, 0.991 and 0.982, respectively.

**Figure 4:**
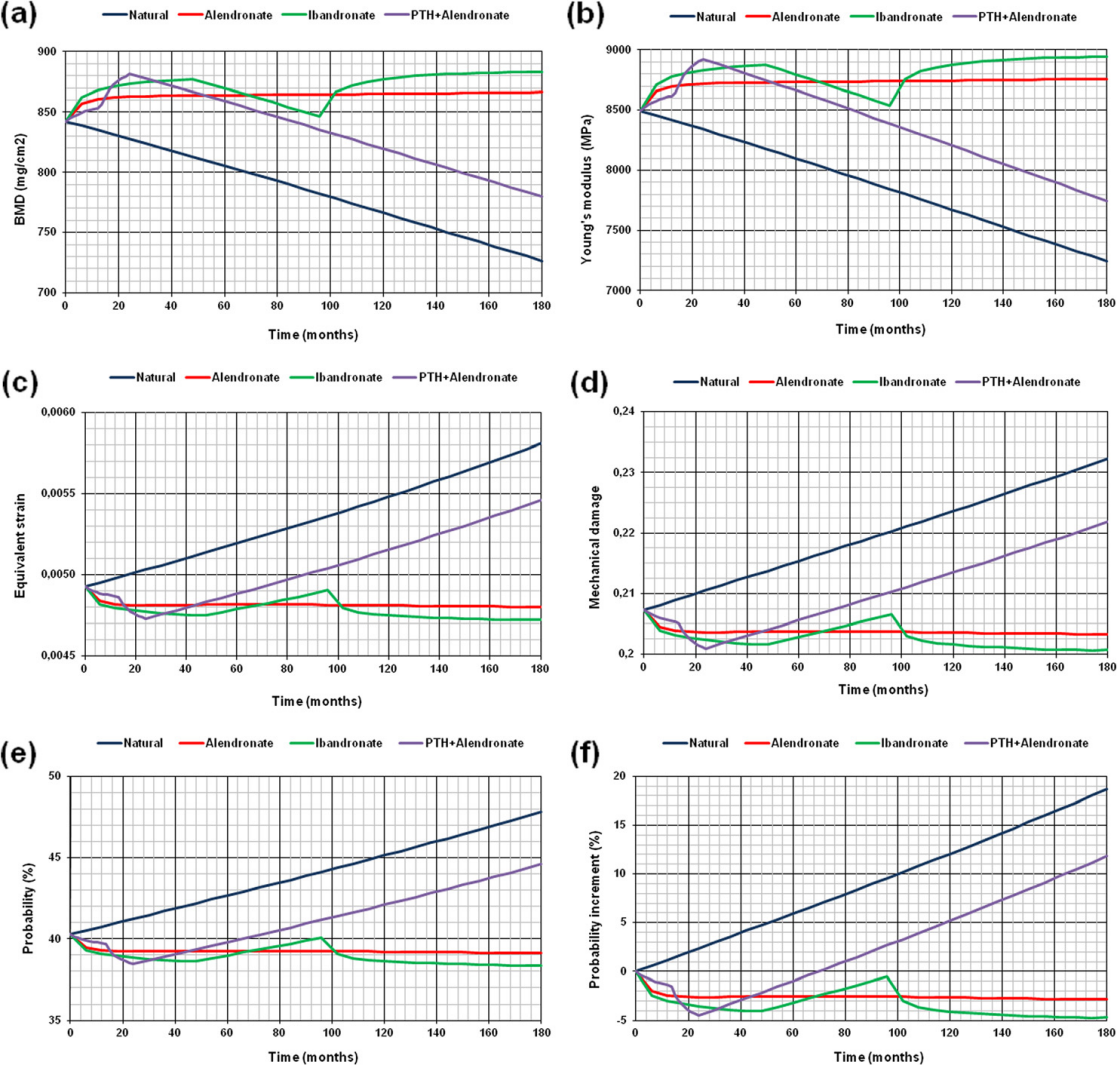
Evolution of different magnitudes at femoral neck: a) BMD; b) Young’s modulus; c) Equivalent strain; d) Mechanical damage; e) Evolution of the fracture probability at femoral neck in different conditions; f) Evolution of the fracture probability increase at femoral neck in different conditions.

Comparisons among various treatments can be drawn regarding different simulations. Figure 4A shows BMD evolution curves under natural conditions and under three therapies (alendronate, ibandronate, and PTH 1–84 plus oral alendronate). As can be seen, patients treated with alendronate showed a significant initial increase in bone density, which remained stable until the end of the study period. Oral ibandronate also leads to a remarkable increase in BMD during the first stage; a progressive decline after treatment interruption (similar to the natural physiological curve), and a new increase with the therapy reintroduction. In opposition, the physiological curve was characterized by a substantial and progressive decline in BMD linked to an increased risk of fracture.

Equivalent comparisons can be made with different parameters. Thus, Figure 4B shows the evolution of Young's modulus in untreated patients and in those under drug therapy (alendronate, ibandronate, and PTH 1–84 plus oral alendronate). Similar patterns to the observed in BMD curves were obtained, according with the direct relationship between the two parameters. Figure 4C shows the evolution of the equivalent strain in subjects under natural conditions and under the same three therapies (alendronate, ibandronate, and PTH 1–84 plus oral alendronate). This equivalent deformation was estimated as a weighted average of the considered bone area. Contrary to previous trends, as bone stiffness decreases the average strain showed a significant increase (as it occurs in the natural history of BMD). In the case of therapies which preserve BMD, and therefore bone stiffness, the average strain value remained stable or even decreases. Finally, Figure 4D illustrates the evolution of mechanical damage under natural conditions and under the three proposed therapies (alendronate, ibandronate, and PTH 1–84 plus oral alendronate). Similar trends to those obtained for strain can be seen. In fact, when the average strains value increases, mechanical damage increases too.

As a final result, the evolutionary curves of fracture probability were obtained from the evolution of mechanical damage. The estimated probability, according to mechanical damage caused by strains, is calculated for the initial patient’s state (Figure 4E). Evolutionary curves of fracture probability increase can be obtained by referencing all results to the initial state (Figure 4F). As can be seen, the fracture probability showed a marked increase in the natural evolution curve, while the curves of the treated patients showed lower degrees of risk, depending on the therapy type.

**Figure 5:**
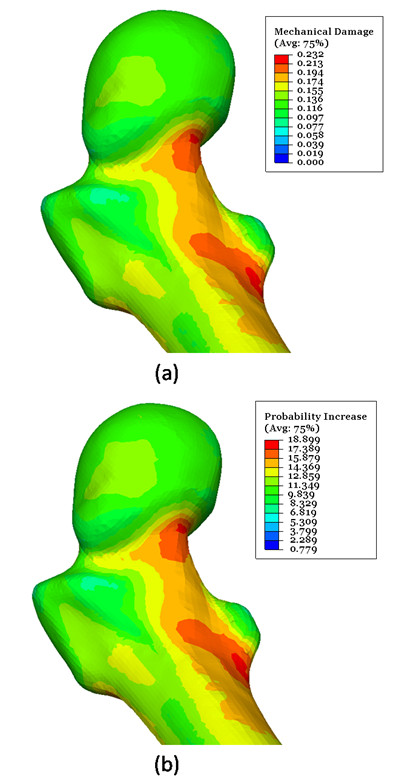
**a)Mechanical damage map for the studied patient(ε**_**0**_ = **0.0015).** Natural evolution (180 months); **b**) Fracture probability increase (%) map for the studied patient (ε_0_ = 0.0015). Natural evolution (180 months).

In addition to previous results, programmed subroutines make it possible to obtain damage and fracture probability maps and to identify high-risk zones of the femoral bone (Figure 5A and b). Femoral neck and intertrochanteric and subtrochanteric areas are the zones at greatest risk, in coincidence with the typical locations of osteoporotic hip fractures. It must be pointed out that Figure 5 doesn’t represent actual fracture zones, but zones with poorer bone mechanical strength due to cumulative damage. The actual fracture zone would be determined according loading scenario (sideway fall, impact, accidental loads, etc.).

## Discussion

A novel method for estimating the risk in osteoporotic patients has been developed. Clinical data (DXA measures) and mechanical magnitudes related to bone strength were combined in this tool. The mechanical properties of bone are updated from BMD values obtained from clinical data of untreated patients and in those under different treatments. The model uses Damage and Fracture Mechanics concepts to evaluate the fracture probability in an evolutionary algorithm.

The model can be used in a personalized way from BMD measurements in each case. The model can contribute to the development of diagnostic tools for detection of early stages of osteoporosis. It may also be helpful for treatment decisions in selected patients. Many studies have been carried out, both in the clinical [9-19] and the simulation fields [20-30]. But a simple and reliable model, useful as a tool for diagnosis and prevention in our daily practice, has not yet been achieved.

Several predictive models can be found in the literature, but statistical models are currently the most reliable [9-12], regardless of mechanical issues involving bone strength estimated for different conditions and ages. The development of new techniques for measuring BMD has focused much of the recent research in the clinical setting, but the mechanical aspects have not been adequately studied [13-16]. In other cases, the improvements have been applied on statistical models previously [18,19]. Only in one published paper [17] a new method derived from DXA measures was developed for bone strength assessment.

Concerning the finite element simulation, and based on previous micromechanical models [20], various methods have been developed, but they are difficult to extrapolate to the scale required to get realistic predictions from different approaches [21,22]. The incorporation of the latest imaging techniques (QCT) and BMD measurement (DXA) [24,27-29] has allowed the improvement of these models. However, most models use standard yield criteria for estimating the risk of fracture [27,28], without considering essential aspects in fracture analysis. Nonetheless, all models assume that bone mineral density is the basic measurement, and it should therefore be used as a benchmark in predicting fracture risk.

Recent works [59] establish a correlation between BMD at femoral neck and fracture risk considering FE analysis and experimental data. Their obtain a von Mises stress distribution with maximum values at femoral neck and subtrochanteric area, just the same zones where the present model predicts the maximum fracture probability.

From the mechanical point of view, the exposition of the bone to cyclic loads of high value in a damaged bone, once the degenerative process is started, decreases its strength over the time and produces a cumulative damage which can lead to a final fracture. It seems apparent that Damage Mechanics and Fracture Mechanics criteria should be incorporated in any model intending to obtain reliable results. In this regard, our model combines all these requirements, and might be useful as a basis for future more sophisticated models.

Moreover, this model enables to incorporate future developments with the same methodology. In the first term, a more accurate bone density distribution, that is, not by Gruen zones but by individual elements in the mesh, could be used. That requires a planned collection of BMD data, by means of DXA or CT scan images. More complex damage models can be added, including mechanical behavior of anisotropic or mixed models, based on both strains and stresses. It would also be possible to include crack growth models fitting to the results of in vitro bone fracture. Finally, a parametric finite element model of the femoral head could be performed including both the loads produced on the bone and the shape and dimensions of a specific patient.

Despite DXA measurements just quantify bone mass and not bone quality, it is widely accepted as a macroscopic indicator of bone strength and stiffness and also that micro-fractures exert an important influence on the mechanical strength of the bone.

Finally, clinical trials are needed to validate the proposed model in order to apply it to the clinical practice helping for treatment decisions.

## Conclusions

A mechanical model based on Damage and Fracture Mechanics and DXA measurements, for predicting the probability of fracture in osteoporotic patients has been carried out. The model represents a first step towards the development of new tools for diagnosis and prevention of osteoporosis. The incorporation of clinical measurements and simulation results will be useful for an individualized monitoring and treatment in specific patients.

## Abbreviations

BMD: Bone mineral density; PTH: Parathyroid hormone; FE: Finite elements; FRAX: Fracture risk assessment tool; WHO: World health organization; DXA: Dual-emission x-ray absorptiometry; NHANES: National health and nutrition examination survey; QCT: Quantitative computed tomography; CT: Computed tomography.

## Competing interests

The authors declare that they have no conflict of interest.

## Authors' contributions

AH and LG conceived the approach of this work. EL and LG conceived and developed the predictive model for fracture probability. AH, ALE and JM contribute with clinical measurements and experience with osteoporotic patients. EL, EI and SP conceived and developed the finite element model and carried out all the simulations. AH and LG coordinated the work between surgeons and engineers. All authors participated in the drawing up of the manuscript, and read and approved the final manuscript.
